# The Autosomal Short Tandem Repeat Polymorphisms Are Potentially Associated with Cardiovascular Disease Predisposition in the Latin American Population: A Mini Review

**DOI:** 10.1155/2023/6152905

**Published:** 2023-11-08

**Authors:** Ana Karina Zambrano, Santiago Cadena-Ullauri, Patricia Guevara-Ramírez, Elius Paz-Cruz, Rafael Tamayo-Trujillo, Viviana A. Ruiz-Pozo, Nieves Doménech, Adriana Alexandra Ibarra-Rodríguez, Aníbal Gaviria

**Affiliations:** ^1^Centro de Investigación Genética y Genómica, Facultad de Ciencias de la Salud Eugenio Espejo, Universidad UTE, Quito, Ecuador; ^2^Instituto de Investigación Biomédica de A Coruña (INIBIC)-CIBERCV, Complexo Hospitalario Universitario de A Coruña (CHUAC), Sergas, Universidad da Coruña (UDC), La Coruña, Spain; ^3^Grupo de Investigación Identificación Genética-IdentiGEN, FCEN, Universidad de Antioquia, Medellín, Colombia; ^4^Laboratorio de Genética Molecular, Centros Médicos Especializados Cruz Roja Ecuatoriana, Quito, Ecuador; ^5^Hemocentro Nacional, Cruz Roja Ecuatoriana, Quito, Ecuador

## Abstract

According to the World Health Organization, cardiovascular diseases (CVDs) are the leading cause of death worldwide across nearly all ethnic groups. Inherited cardiac conditions comprise a wide spectrum of diseases that affect the heart, including abnormal structural features and functional impairments. In Latin America, CVDs are the leading cause of death within the region. Factors such as population aging, unhealthy diet, obesity, smoking, and a sedentary lifestyle have increased the risk of CVD. The Latin American population is characterized by its diverse ethnic composition with varying percentages of each ancestral component (African, European, and Native American ancestry). Short tandem repeats (STRs) are DNA sequences with 2-6 base pair repetitions and constitute ~3% of the human genome. Importantly, significant allele frequency variations exist between different populations. While studies have described that STRs are in noncoding regions of the DNA, increasing evidence suggests that simple sequence repeat variations may be critical for proper gene activity and regulation. Furthermore, several STRs have been identified as potential disease predisposition markers. The present review is aimed at comparing and describing the frequencies of autosomal STR polymorphisms potentially associated with cardiovascular disease predisposition in Latin America compared with other populations.

## 1. Introduction

According to the World Health Organization, cardiovascular diseases (CVDs) are the leading cause of death worldwide across nearly all ethnic groups [1, 2]. It is estimated that 17.9 million people die each year due to these diseases, accounting for 31% of deaths globally [2]. CVDs have been associated with the complex interplay of genetic, epigenetic, and environmental factors [3–7]. For instance, inherited cardiac conditions comprise a spectrum of diseases affecting the heart, including abnormal structural features and functional impairments. In most cases, these conditions are transmitted in an autosomal dominant manner, potentially affecting multiple generations within a family due to the presence of monogenic or polygenic variants [2, 8]. Moreover, these diseases are influenced by a combination of modifiable and nonmodifiable risk factors. For example, the modifiable risk factors include physical inactivity, tobacco use, high low-density lipoprotein cholesterol, and obesity [9, 10]. In contrast, nonmodifiable risk factors encompass race, gender, aging, and family history [9].

The overall prevalence of CVD worldwide is 3%; some can cause sudden morbidity or mortality in young people. One example is hypertrophic cardiomyopathy, which is generally not diagnosed prematurely due to a lack of symptoms and is one of the most common cardiovascular diseases reported worldwide, with a prevalence of 1 in every 500 individuals [11, 12]. Similarly, familial hypercholesterolemia, a genetic disorder that can lead to morbidity or premature death from atherosclerotic cardiovascular disease, has been found worldwide, representing a prevalence of 1 in every 200 to 250 individuals. It is estimated that 34 million people have undiagnosed familial hypercholesterolemia [13, 14].

In Latin America, cardiovascular diseases (CVDs) are the leading cause of mortality within the region [15]. Moreover, the challenge of limited access to healthcare, which is particularly prevalent in Latin America, has further augmented the problem [16]. For example, Leitão et al. [17] reported that 27.7% of deaths in Brazil were attributable to CVD. In Argentina, this figure climbs to 30% of the total deaths [18]. In Peru, CVD accounted for 20% of deaths in 2016 [19], a statistic similar to Mexico, where 22.7% of deaths in 2017 were CVD-associated [20]. In Colombia, 28% of deaths are related to CVD [21], and finally, in Ecuador, the percentage of CVD-associated deaths is 26.4% [22].

The Latin American population is a heterogeneous mix of ethnic backgrounds; for example, a published study using informative ancestry insertion and deletion markers in a sample of the Ecuadorian population of the three continental regions of Ecuador that self-identified as mestizo revealed that the ancestral component was as follows: 59.6% Native American, 11.6% African, and 28.8% European [23]. Similarly, Pena et al. [24] reported extensive admixture in the Brazilian population, with an average European ancestry of 0.70, an African component of 0.18, and an Amerindian component of 0.12. Although the percentage of each ancestral component may vary, Latin America is a trihybrid region composed of African, European, and Native American elements [23–25].

Short tandem repeats (STRs) are DNA sequences with 2-6 base pair repetitions and constitute ~3% of the human genome [26, 27]. These regions are one of the most polymorphic across the human genome [28], and the number of repetitions is variable depending on the individual, offering the possibility of using it for forensic purposes [26, 29].

Moreover, a notable variation in allele frequencies exists between different populations [29]. Previous studies have located STRs in noncoding regions of the DNA [26]. Nevertheless, increasing evidence suggests that simple sequence repeat variations, such as STRs, are not randomly distributed across introns, UTRs, and protein-coding regions [30–32]. This evidence suggests that they may be critical for proper gene activity and regulation [26].

Furthermore, several STRs have been described as disease predisposition markers [26, 31, 32]. Interestingly, while these STRs have been phenotypically linked to a specific population, they could also serve as phenotype indicators for diverse populations based on the presence of these STRs, independently of their frequency.

Additionally, numerous studies have identified forensic STRs as genetic risk factors for cardiovascular diseases [26, 33–37]. For instance, the alleles 26 and 27 of the FGA marker located in 4q28 within the third intron of the human alpha fibrinogen gene have been associated with ischemic stroke, carotid artery intima–medial thickness, and other cardiovascular events [34, 35]. The allele 28.2 of the D21S11 marker, situated in 21q21.1, has been linked to coronary heart disease [33].

TPOX is located in 2p25.3, in intron 10 of the human thyroid peroxidase gene; its genotypes 9 and 12, in their homozygous form, can act as risk factors for venous thromboembolism [26, 36]. Furthermore, TH01, a marker located in 11p15.5, specifically in intron 1 of the human tyrosine hydroxylase gene, has been associated with various neurological, psychiatric, and cardiovascular diseases [26]. For example, TH01-9.3 was the most frequent allele observed in sudden infant death syndrome (SIDS) [38].

Finally, the STR vWA (von Willebrand factor type A), located in 12p13.31 within the 40th intron, has been analyzed by Meraz-Ríos et al. [36]. This study showed a correlation between vWA-18 and venous thrombosis . Similarly, another study linked vWA with von Willebrand's disease [37].

The present review is aimed at comparing and describing the frequencies of autosomal STR polymorphisms potentially associated with cardiovascular disease predisposition in Latin America.

## 2. Short Tandem Repeats Associated with Cardiovascular Disease Predisposition

A complete literature search was conducted to identify the alleles associated with an increased risk of cardiovascular diseases. The search included databases such as PubMed and Google Scholar and employed specific search terms including “STR,” “cardiovascular disease,” and “predisposition.” Eight alleles in five Combined DNA Index System (CODIS) STR loci were identified as having a potential role in CVD predisposition. These alleles were TPOX-9, TPOX-12, FGA-26, FGA-27, TH01-9.3, TH01-10, vWA-18, and D21S11-28.2.

## 3. Frequency Data of Short Tandem Repeats Associated with Cardiovascular Disease Predisposition in Latin American, European, Asian, and African Populations

The frequency of each allele is shown in Table 1.

## 4. Correlation between Cardiovascular Disease Predisposition and Short Tandem Repeats

Cardiovascular diseases are complex disorders with genetic, epigenetic, and environmental interactions [57, 58]. A complete literature search was conducted to identify the alleles that have been reported to be involved in the risk of cardiovascular diseases. The search selected five CODIS STR loci for analysis, leading to the identification of eight specific alleles. The allele frequencies are presented in Table 1.

**Figure 1:**
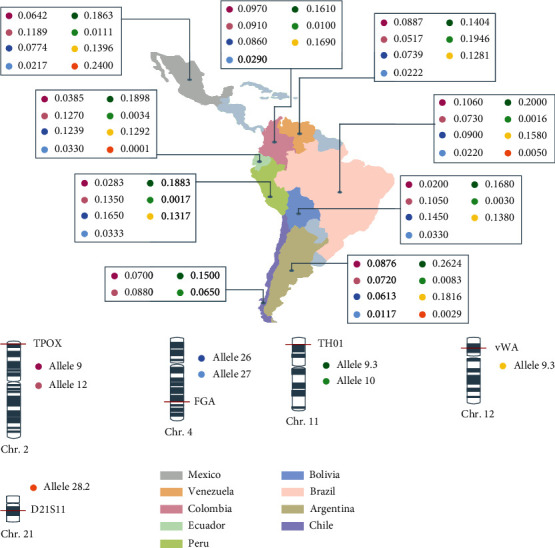
CVD predisposition-associated alleles in Latin American countries. Chromosomal location and allelic frequency are displayed.

**Figure 2:**
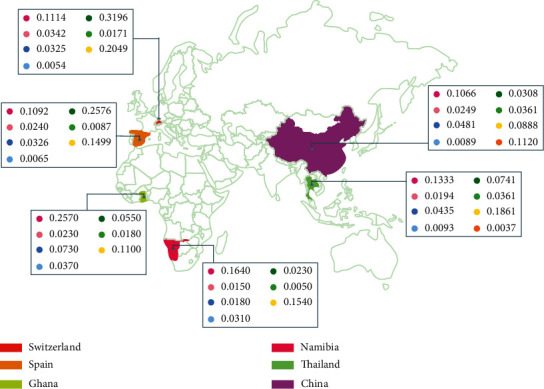
CVD predisposition-associated alleles in the world. The allele frequency of each country is displayed on the map.

The potential CVD predisposition allele chromosomal location is presented in Figure 1, along with a map identifying the Latin American countries included in this review with each country's allele frequency. Moreover, Figure 2 represents a map of the European, Asian, and African countries selected and each allele frequency.

### 4.1. FGA

The FGA gene, located on chromosome 4, encodes the coagulation factor fibrinogen *α*. Moreover, the studied STR is located within the third intron. It is a tetranucleotide repeat with the sequence [TTTC]_3_TTTTTTCT[CTTT]_n_CTCC[TTCC]_2_ [59]. Specific variants within this gene have been associated with venous thromboembolism [60], ischemic stroke [35], fibrinogen concentration, carotid artery intima-media thickness, and risk of incident myocardial infarction [34]. Additionally, FGA single nucleotide polymorphisms (SNPs) have been correlated with elevated fibrinogen levels, which can potentially lead to inflammatory states associated with CVD [61, 62].

Furthermore, alleles 26 and 27 showed a possible association with thrombosis [36]. According to Meraz-Ríos et al. [36], the allelic frequency for FGA-26 in cases with thrombosis was 0.1243, while the allelic frequency of FGA-27 was 0.0876 within the Mexican population.

It is noteworthy that the allelic frequency of FGA-26 is higher in Peru, a country where CVD accounts for 20% of deaths, reaching 0.1650 [19, 42]. On the other hand, for the FGA-27 allele, Ghana exhibits the highest frequency with 0.0370; in this country, 14.5% of total deaths are attributed to CVD [56, 63].

### 4.2. D21S11

The D21S11 locus is located on chromosome 21 (21q21.1), and more than nine genes are found within this region [64]. The sequence of this locus is characterized by [TCTA] and [TCTG] repeats [59]. Among the genes nearby, TMPRSS15 (Transmembrane Serine Protease 15) stands out, with annotations related to serine-type endopeptidase activity and scavenger receptor activity [65]. GART (Phosphoribosylaminoimidazole Synthetase), another gene found within this region, has annotations related to methyltransferase activity and phosphoribosylamine-glycine ligase activity [66], among others. Furthermore, GART has been linked to an increased diastolic interval in *Drosophila*. It is important to highlight that this region is conserved in humans, which could indicate concordant functions [67].

Similarly, the gene CRYZL1, also found within this locus, has been correlated with coronary artery disease [68]. Hui et al. [33] described an association between the presence of the D21S11-28.2 allele and a significant age difference in coronary heart disease (CHD). Notably, the highest D21S11-28.2 frequency was reported in the Mexican population (0.240) [39]. The CVD death rate in the Mexican population is similar to the Peruvian population, accounting for 20% of total deaths [39, 69].

### 4.3. TPOX

The TPOX locus is located on chromosome 2 (2p25.3) within the intronic sequence of the human thyroid peroxidase (TPO) gene. Its sequence is [AATG] [59]. Reports have suggested that the activity of the thyroid peroxidase enzyme is associated with coagulation abnormalities and vascular endothelial dysfunctions, potentially contributing to an increased thromboembolic potential, a factor linked to venous thromboembolism [36, 70, 71].

Moreover, the TPO gene has been correlated with congenital hypothyroidism [72], a condition that can lead to a specific type of cardiomyopathy [73, 74]. TPOX-9 and TPOX-12 were related to thrombosis risk in a Mexican population, showing a frequency of 0.3305 and 0.2062 in thrombotic cases, respectively [36]. Ghana showed the highest TPOX-9 frequency, with 0.2570 [56], whereas Peru had the highest TPOX-12 frequency (0.1350) [42].

### 4.4. TH01

The short tandem repeat marker TH01 is situated on chromosome 11 (11p15.5), within the human tyrosine hydroxylase gene, characterized by its sequence [TCAT] [59]. The encoded protein regulates gene expression and catecholamine production (like noradrenaline or adrenaline), which are pivotal in blood pressure regulation [72].

Moreover, variants in this gene have been associated with alterations in blood pressure and human autonomic function [75]. Notably, Courts and Madea [76] found a correlation between the allele TH01-9.3 and sudden infant death syndrome in Bonn, Germany. Additionally, both TH01-9.3 and TH01-10 have been related to hypertension [77].

Interestingly, TH01-9.3 frequency was the highest in Switzerland [38], a country where CVD accounts for 35% of total deaths [78, 79]. On the other hand, the frequency of TH01-10 (0.1946) was the highest in Venezuela [46], where CVD contributes to 17.07% of total deaths in the country [80].

### 4.5. vWA

Lastly, the locus vWA is located within chromosome 12 on the von Willebrand factor (VWF) gene. VWF is a glycoprotein synthesized by vascular endothelial cells and platelets [81, 82]. Its sequence is [TCTA] with [TCTG] and [TCCA] inserts [59]. VWF is essential for homeostasis, promoting thrombosis by platelet adhesion and aggregation, and clinically acts as a biomarker of cardiovascular disease [83].

Furthermore, VWF is pivotal in promoting inflammation by recruiting platelets and leukocytes while modulating vascular tissue [83]. Individuals carrying the vWA-18 allele showed a high risk of venous thromboembolism in the Mexican population [36]. Noteworthily, Switzerland has the highest vWA-18 (0.2049), and as stated before, CVD accounts for 35% of the total deaths in the country [50, 51, 78].

### 4.6. Genes Commonly Associated with Cardiovascular Diseases

Genes commonly associated with CVD include *PCSK9*, *APOB*, *CETP*, *LPL*, and *MYBPC3* [84, 85]. For instance, mutations in the genes *PCSK9* and *APOB* have been specifically related to familial hypercholesterolemia. In the case of *PCSK9*, variants can lead to a decrease in the number of low-density lipoprotein (LDL) receptors, while *APOB* variants may disrupt the binding between LDL and its receptors [84, 85].

## 5. Population Comparison

Latin American populations are composed of African, European, and Native American elements [23–25]; although the percentage of each component may vary, all the countries in the region have gone through similar historical processes [86]. For example, between Ecuador and Peru, almost all alleles have similar frequencies, with standard deviations from 0.029 to 0.00021, reflecting a shared history, even evidenced in STR frequencies [41, 42].

Interestingly, the Peruvian population had the highest frequency in two alleles associated with CVD (FGA-26 and TPOX-12) [42]; however, Peru reported the lowest percentage of CVD deaths [19]. Similarly, Ghana has the highest TPOX-9 and FGA-27 allele frequencies [56] and reports one of the lowest CVD-correlated death rates. On the other hand, Switzerland had the highest TH01-9.3 and vWA-18 allele frequencies, in concordance with the CVD-death rate [50, 51, 78, 79]. It is important to emphasize that even though the studies by Meraz-Rios et al. [36], Hui et al. [33], and Courts et al. [76] suggested a potential association between STRs and cardiovascular disease predisposition, none of the relationships mentioned in this review provide evidence of a direct causal relationship. More case-control studies must be conducted to elucidate their role in CVD.

Information regarding the impact of STRs on cardiovascular disease remains scarce, making it challenging to stablish an association between STR frequencies and specific populations. The present manuscript describes the association between 8 alleles and CVD predisposition, highlighting that the presence of an STR association with a phenotype does not necessarily mean causation [87].

Furthermore, when comparing allele frequencies, statistically significant differences have been observed. However, these disparities may be explained by evolutionary processes such as population substructure and genetic distances between populations [88]. Therefore, further studies are needed to comprehensively understand the correlation between STRs and CVD predisposition.

## 6. Conclusion and Future Perspectives

The present review compares and describes the frequency data of STRs associated with CVD predisposition, as reported in previous studies. Moreover, several variants within different genes have been directly associated with a cardiovascular phenotype. In this case, the described eight alleles could be potential candidate STR markers to identify a predisposition to cardiovascular disease. However, case-control studies should be performed to elucidate if there is a significant association between the STR alleles and the risk of developing cardiovascular disease.

## Figures and Tables

**Table 1:** Frequency of potentially associated CVD predisposition alleles in nine Latin American and two European, Asian, and African countries.

Marker	TPOX		FGA		TH01		vWA	D21S11	Reference
Allele	9	12	26	27	9.3	10	18	28.2	
Mexico	0.0642	0.1189	0.0774	0.0217	0.1863	0.0111	0.1396	**0.2400**	[39]
Colombia	0.0970	0.0910	0.0860	0.0290	0.1610	0.0100	0.1690		[40]
Ecuador	0.0385	0.1270	0.1239	0.0330	0.1898	0.0034	0.1292	0.0001	[41]
Peru	0.0283	**0.1350**	**0.1650**	0.0333	0.1883	0.0017	0.1317		[42]
Brazil	0.1060	0.0730	0.0900	0.0220	0.2000	0.0016	0.1580	0.0050	[43]
Argentina	0.0876	0.0720	0.0613	0.0117	0.2624	0.0083	0.1816	0.0029	[44]
Bolivia	0.0200	0.1050	0.1450	0.0330	0.1680	0.0030	0.1380		[45]
Venezuela	0.0887	0.0517	0.0739	0.0222	0.1404	**0.1946**	0.1281		[46]
Chile	0.0700	0.0880			0.1500	0.0650			[47]
Spain	0.1092	0.0240	0.0326	0.0065	0.2576	0.0087	0.1499		[48, 49]
Switzerland	0.1114	0.0342	0.0325	0.0054	**0.3196**	0.0171	**0.2049**		[50, 51]
China	0.1066	0.0249	0.0481	0.0089	0.0308	0.0361	0.0888	0.1120	[52, 53]
Thailand	0.1333	0.0194	0.0435	0.0093	0.0741	0.0361	0.1861	0.0037	[54]
Namibia	0.1640	0.0150	0.0180	0.0310	0.0230	0.0050	0.1540		[55]
Ghana	**0.2570**	0.0230	0.0730	**0.0370**	0.0550	0.0180	0.1100		[56]
Standard dev.	0.0585	0.0425	0.0434	0.0114	0.0861	0.0493	0.0311	0.0981	

The standard deviation of each allele frequency is presented at the bottom of the table. The highest frequency of each allele is in bold.

## Data Availability

All data used to support the findings of this study are included in the article.
